# Selective time-dependent changes in activity and cell-specific gene expression in human postmortem brain

**DOI:** 10.1038/s41598-021-85801-6

**Published:** 2021-03-23

**Authors:** Fabien Dachet, James B. Brown, Tibor Valyi-Nagy, Kunwar D. Narayan, Anna Serafini, Nathan Boley, Thomas R. Gingeras, Susan E. Celniker, Gayatry Mohapatra, Jeffrey A. Loeb

**Affiliations:** 1 University of Illinois at Chicago, Chicago, IL 60612 USA; 2 Lawrence Berkeley National Laboratory, Berkeley, CA 94720 USA; 3 University of California, Berkeley, CA 94720 USA; 4 Cold Spring Harbor Laboratory, Cold Spring Harbor, NY 11724 USA

**Keywords:** Genetics of the nervous system, Neurological disorders

## Abstract

As a means to understand human neuropsychiatric disorders from human brain samples, we compared the transcription patterns and histological features of postmortem brain to fresh human neocortex isolated immediately following surgical removal. Compared to a number of neuropsychiatric disease-associated postmortem transcriptomes, the fresh human brain transcriptome had an entirely unique transcriptional pattern. To understand this difference, we measured genome-wide transcription as a function of time after fresh tissue removal to mimic the postmortem interval. Within a few hours, a selective reduction in the number of neuronal activity-dependent transcripts occurred with relative preservation of housekeeping genes commonly used as a reference for RNA normalization. Gene clustering indicated a rapid reduction in neuronal gene expression with a reciprocal time-dependent increase in astroglial and microglial gene expression that continued to increase for at least 24 h after tissue resection. Predicted transcriptional changes were confirmed histologically on the same tissue demonstrating that while neurons were degenerating, glial cells underwent an outgrowth of their processes. The rapid loss of neuronal genes and reciprocal expression of glial genes highlights highly dynamic transcriptional and cellular changes that occur during the postmortem interval. Understanding these time-dependent changes in gene expression in post mortem brain samples is critical for the interpretation of research studies on human brain disorders.

## Introduction

Research in animal models that aim to understand fundamental disease processes and develop new treatments for human disorders often fail to translate back to humans in clinical trials^1–3^. Human tissue studies are therefore often used to try to improve this poor translational track record and to validate future therapeutic targets. Nowhere is this approach more desperately needed than for the human brain where post-mortem tissue repositories for a large number of neurological and psychiatric disorders already exist. In some brain disorders, including epilepsy, freshly isolated tissues can be obtained as part of a patient’s surgical treatment and used for research. Co-registration of these tissues with their corresponding activity measured by long-term intracranial EEG allows the separation of high versus low epileptic activity brain regions. This provides a unique opportunity to ask what is different between high and low electrical activity brain regions and to identify genes, proteins, and small molecules that are differentially expressed between them^4^. For a majority of neuropsychiatric disorders including Alzheimer’s disease, Autism, and Schizophrenia, only postmortem tissues are available^5^. Given the importance of these studies we examined the fidelity of overall gene expression between fresh and postmortem human brain tissues for a number of brain disorders with a central focus on activity-dependent genes that are likely involved in higher cognitive human brain function. In order to understand the dynamics of these transcriptional differences, we also simulated the post-mortem interval (PMI) on surgically isolated fresh human brain tissue maintained at room temperature from 0 to 24 h using high throughput RNA sequencing (RNA-seq) paired with histological examination^6^.

## Results

### Transcriptional integrity of fresh human brain tissue

As a means to assess the fidelity of RNA transcription from fresh human brain tissue, we performed RNAseq on four human neocortical gray matter tissues with high and low brain activity regions from each of two patients who underwent epilepsy surgery. This was achieved by localizing each block of tissue precisely to electrode locations from intracranial in vivo recordings to identify areas of high and low epileptic brain activity as described^7–9^. Histopathological examination of these four tissues showed no abnormalities. The exact pattern of differential gene expression between these areas for these four samples by RNAseq matched what we have seen both from these same samples and from many others using cDNA microarrays^9^.

**Figure 1:**
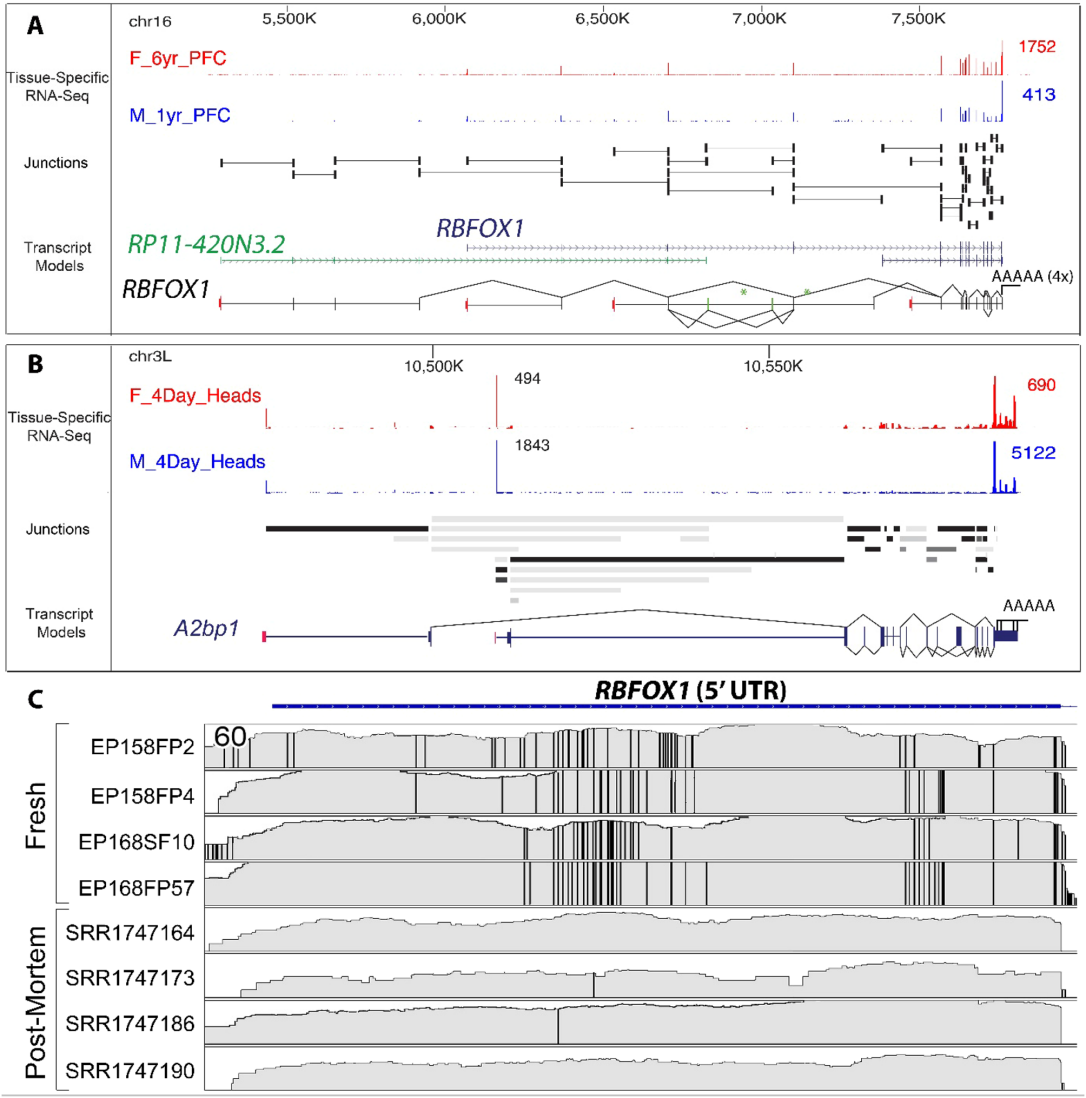
Ultra-complex structure of the *RBFOX1* gene. (**A**,**B**) Human/Fly splicing complexity analysis: for each RNA detected in the RNAseq results of patients EP158 and EP168, the lower and upper bounds of divergence between human and fly Drosophila is computed using our GRIT algorithm. The *RBFOX1* gene was selected for illustration of this due to its complexity, evolutionary conservation, and relevance to human brain disease. (**C**) RNAseq alignment of *RBFOX1* RNAs from 4 fresh samples (EP158: electrodes FP2 & FP4, EP168: electrodes SF10 & FP57) and 4 postmortem samples (SRR1747164, SRR1747173, SRR1747186, SRR1747190) show a significant departure from the reference genome (vertical bars) that reveals extensive gene editing in fresh samples that were not seen in the postmortem samples (PMI = 29 h ± 2.6 h).

As a first step to see the effect of the postmortem interval on human brain gene expression, we compared the transcriptional diversity of RNAseq gene expression between fresh human brain samples to 4 postmortem brain samples from healthy individuals with an average PMI of 29 h (± 2.6 h)^10^. Fresh human brain showed marked transcriptional diversity with a collection of 167 genes each capable of producing more than 1000 transcript isoforms (Supplemental_Table_S1.xlsx). This type of ultra-complex splicing appears to be highly conserved evolutionary and has been previously reported in the nervous system of the fruit fly *Drosophila melanogaster*^11^. An example of this transcriptional diversity is illustrated in Fig. 1A,B that shows the remarkable conservation of the ultra-complex structure of the *RBFOX1* gene and of its orthologue *a2bp1* in the fly brain. We next compared RBFOX1 splicing and editing complexity between the fresh tissue and postmortem human brain samples. While qualitatively similar to the fresh human tissue, the expression of RBFOX1 in postmortem samples showed a wide reduction in fidelity from well preserved to almost completely absent from sample to sample. 5′UTR transcripts of *RBFOX1* showed a complex pattern of potential RNA editing in the fresh tissue that was not seen in postmortem samples (Fig. 1C). A total of 17 departures from the reference genome were present in all fresh tissues but not present (or at a very low frequency) in the postmortem samples. These differences can be due in part by RNA deamination by the ADAR enzyme from adenosine to inosine as 8 of these misalignments were a change from A-to-G, with the presence nearby of four editing motifs A, B or C^12^. The remaining 9 other misalignments consistent across the fresh samples (6 U-to-G, 2 U-to-C and 1 A-to-C) could be due to non-canonical post-transcriptional RNA editing^13^. These mismapped alignments could also be caused by reads from a similar genomic duplicated region expressed in fresh samples but not in postmortem samples^14^. Taken, together this focused example shows a remarkable difference in the transcriptional complexity of an important brain gene in fresh versus post-mortem human brain.

### Selective loss of specific gene populations in neuropsychiatric disorders

In addition to detailed splicing information on the *RBFOX1* gene, we examined the overall genomic coverage of fresh versus postmortem human brain. We examined 18,064 genes expressed in at least one replicate in both sample types and performed a two-group differential expression analysis between fresh and postmortem tissues. The goal was to determine whether there are specific populations of genes downregulated in postmortem tissues or whether transcripts are simply, randomly reduced from non-specific RNA degradation during the postmortem interval.

**Figure 2:**
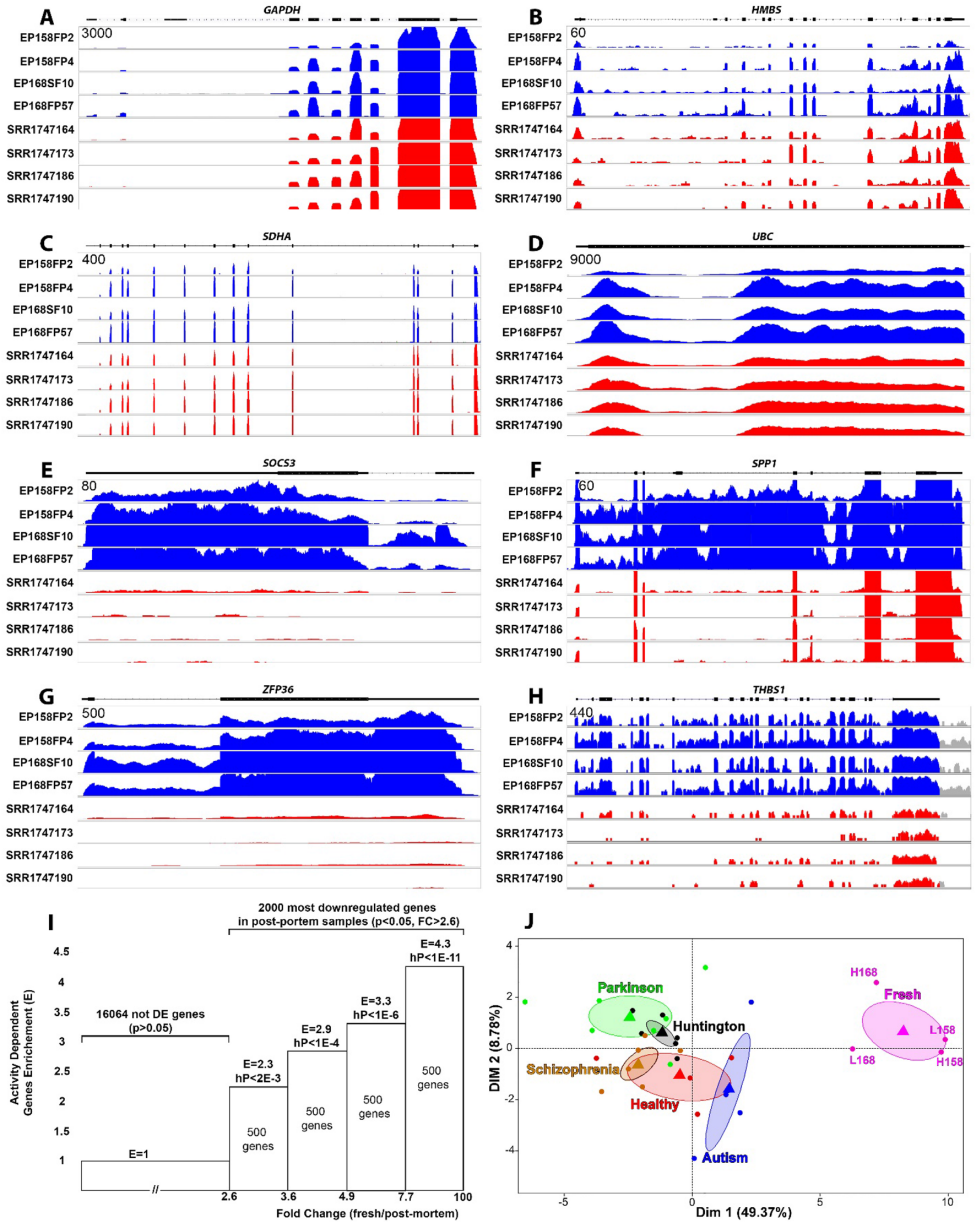
Selective loss of activity-dependent genes in postmortem human brain. (**A**–**H**) The RNAseq-derived gene expression coverage from four fresh human cortex tissues (EP158FP2, EP158FP4, EP168SF10 and EP168FP57) are shown in blue and compared to healthy postmortem cortex samples (SRR1747164, SRR1747173, SRR1747186 and SRR1747190) shown in red. While housekeeping genes including *GAPDH*, *HMBS*, *SDHA* and *UBC* appear relatively unaffected, the activity-dependent genes *SOCS3*, *SPP1*, *ZFP36* and *THBS1* showed a significant reduction in exon-specific transcript levels in postmortem samples (p < 0.05). For each gene, the y-axis is the same for each sample with the scale in the upper left corner. (**I**) We cross-referenced 2000 of the most downregulated genes in postmortem samples compared to fresh samples and ordered them based on their fold change (expression of the gene in fresh tissue/expression of the gene in postmortem sample) into four groups of 500 genes each. For each group, we computed the enrichment in activity-dependent genes. The hypergeometric analysis of the distribution of activity-dependent genes shows their expression is significantly reduced, more than four times what is expected by chance in the 500 most downregulated genes of the postmortem samples, *E* Enrichment, *hP* hypergeometric p-value. (**J**) A PCA of the 500 activity-dependent genes with the greatest decrease in expression in the postmortem samples highly separated all the fresh tissues samples from the postmortem samples, independent of the degree of epileptic activity. Each dot corresponds to a given sample, the barycenters are represented by triangles and the ellipses show the 80% confidence interval for the position of the barycenter. L158, L168, H158, H168 correspond to the four fresh samples from patient EP158 and EP168, ‘L’ corresponds to low activity brain regions and ‘H’ corresponds to the high activity brain regions.

Figure 2A–D shows the expression and splicing patterns of some of the most widely used ‘housekeeping’ genes (*GAPDH*, *HMBS*, *SDHA*, and *UBC*)^15,16^ that have little to no degradation with exon expression patterns are virtually identical between fresh and postmortem brain. However, other genes, similar to what we observed for *RBFOX1,* were significantly degraded. As our group has a major interest in brain activity-dependent gene expression, we compared human brain regions recorded in vivo with high versus low epileptic activities. We specifically looked at a well-described subgroup of differentially expressed activity-dependent genes^9^. Figure 2E–H shows examples of exon stability for these activity-dependent genes. There is a disproportionate loss of exon expression in postmortem tissues compared to the fresh tissues of *RGS1*, *SOCS3*, *THBS1* and *ZFP36*. Furthermore, fresh human brain appeared to have more RNAseq reads overlapping introns than postmortem samples (e.g. *SPP1* and *SOCS3*). The increase in the number of introns in the fresh tissues also suggests an abundance of pre-mRNA transcripts that could be related to RNA editing.

Hypergeometric analysis of the 2000 most reduced genes in postmortem samples (p-value < 0.05, ratio of expression in fresh tissue / expression in postmortem tissue > 2.6) showed that a large portion of these genes overlap with activity-dependent genes, suggesting that activity-dependent genes are more susceptible to postmortem RNA degradation than housekeeping genes (Fig. 2I)^8,9,17^. This reduction was non-random and the genes that showed the greatest decrease in expression in postmortem samples were also the most enriched in electrically active brain regions (Fig. 2I). In fact 500 of the most reduced genes in postmortem samples showed an enrichment in activity-dependent genes that was more than 4 times greater than would be expected by chance (enrichment of 4.25 with an hypergeometric p-value < 1E−11).

As brain activity-dependent human genes are of great importance in human neuropsychiatric disorders we also examined the expression of these genes to postmortem RNAseq databases from patients suffering from various neurological and psychiatric disorders (Table 1). Datasets were chosen based on similarities in tissue processing and RNAseq methodology to our own protocol. We performed a PCA (Principal Component Analysis) of our fresh brain compared to postmortem brain from healthy, Parkinson’s, Schizophrenia, Huntington’s, and Autism brains for the top 500 brain activity-dependent genes that showed the greatest reduction in the healthy postmortem samples. The PCA revealed a significant separation between the 4 fresh samples and the postmortem samples, independent of whether or not the fresh tissue was from epileptic (high activity, H) or non-epileptic (low activity, L) brain regions (Fig. 2J). This further demonstrates a selective reduction of activity-dependent genes in postmortem brain independent of whether the underlying tissue is electrically active or not.

**Table 1:** Samples and RNAseq data used in this study.

Sample types	Number of samples	Age (years)	Sex (% male)	PMI (hours)	Brain tissue	References
Epilepsy	4	4 ± 3	50%	0	Grey matter	Present study
Autism	4	5 ± 1	50%	Unknown	Grey matter enriched	^18^
Healthy controls	4	56 ± 13	100%	29 ± 2.6	Grey matter	^10^
Huntington’s disease	6	59 ± 10	100%	16 ± 9	Grey matter	^10^
Parkinson’s disease	7	76 ± 9	100%	20 ± 13	Prefrontal cortex	^19^
Schizophrenia	6	55 ± 5	67%	30.3 ± 5.3	Prefrontal cortex	^20^
Epilepsy	7	–	–	0 to 24	Grey matter	Used to simulate the PMI

### Simulating the postmortem interval as a means to understand selective gene loss

The sudden removal of brain tissue from a living person in many ways mimics a catastrophic event that occurs with a hypoxic brain injury or a traumatic death with exsanguination. The human brain has high energy needs, estimated to be 10 times that of other tissues^21^. As a means to understand how the postmortem interval selectively affects some genes and not others in human neocortex, we performed RNAseq and histological analyses in cortical brain tissue as a function of time from 0–24 h at 24 °C in order to simulate a postmortem interval. Neuropathological examination of the tissue used for this study showed a normal-appearing cortical pattern with no histopathologic abnormalities. RNAseq analysis showed a loss of brain activity-dependent genes that were 3-times more prone to be degraded than expected by chance compared to more stable housekeeping genes (Table 2). The threshold to detect activity-dependent genes was related to the probability of being affected by the PMI. The higher the relative expression of the brain activity gene, the more it was enriched in the population of genes affected by the PMI. These findings confirm that genes involved in brain activity are more prone to degradation during the PMI.

**Table 2:** Brain activity-dependent genes are enriched in the population of genes affected by the simulated PMI.

FC (FDR 1%)	Total	Regulation	PMI-stable	PMI-not-stable
|1.5|	524	Up or down	99	356 (E = 4.1)
|1.4|	863	Up or down	182	543 (E = 3.4)
|1.3|	1998	Up or down	580	1065 (E = 2.1)
			Average enrichment	**E = 3.2**

We tested three groups of genes using fold change (FC) cutoffs of 1.3, 1.4 and 1.5 (corresponding to the absolute values of the ratio of expression of high brain activity genes divided by the expression of low activity brain genes). The significance used was FDR ≤ 1%. A total of 6754 genes (43%) were detected as stable, 5947 (38%) were identified as not stable from 0 to 24H simulated PMI. We then calculated the enrichment in brain activity-dependent genes inside each group and labeled these as ‘PMI not-stable’. Enrichment was calculated as the ratio between the quantity of brain activity-dependent genes that were not stable as a function of simulated PMI divided by the quantity of brain activity-dependent genes that were stable.

*E* Enrichment.

**Figure 3:**
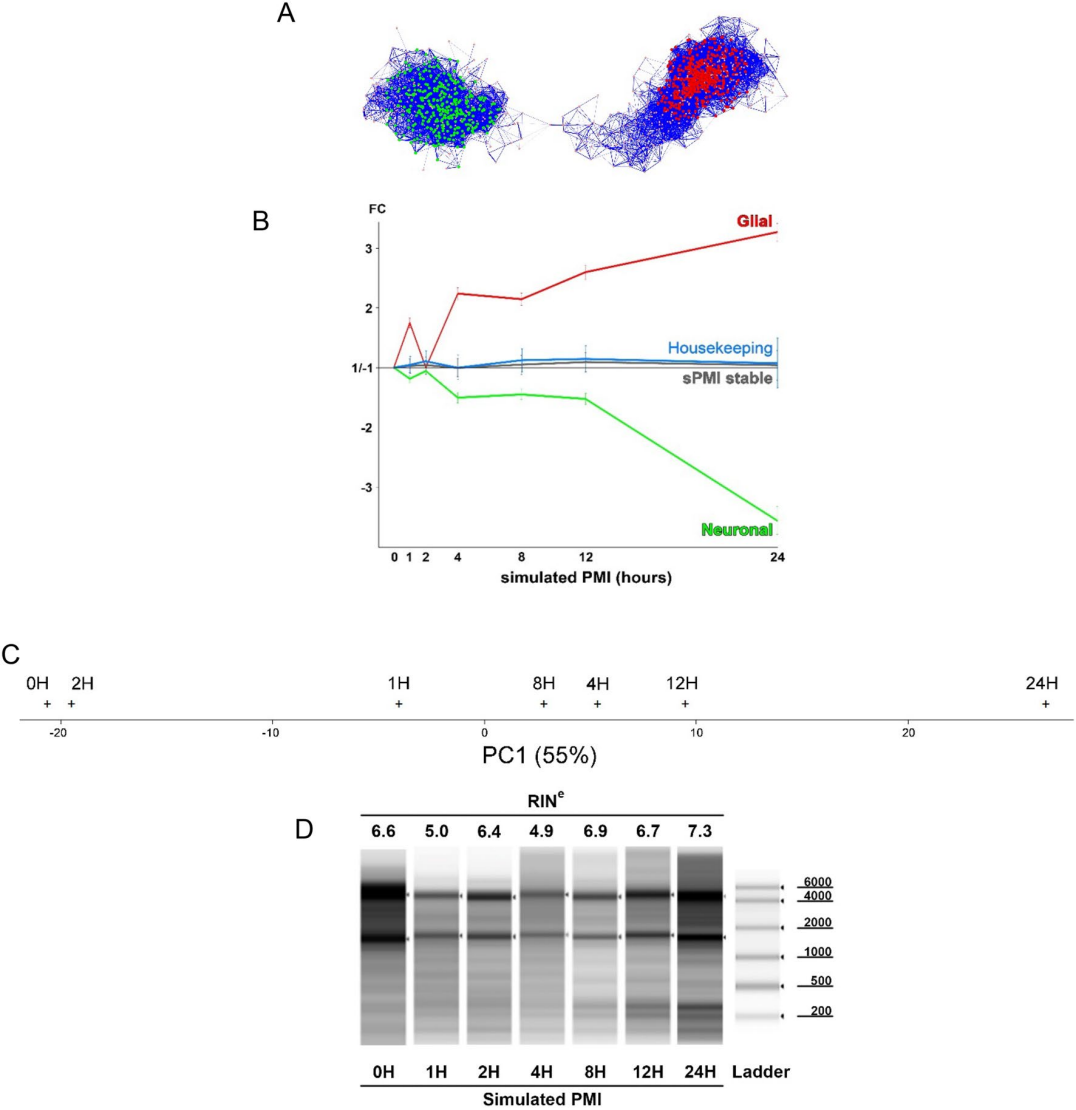
Gene clustering of brain activity-dependent genes reveals a reciprocal loss of neuronal genes inversely proportional to a rise in glial genes. (**A**) Analysis of the expression of 1998 activity-dependent genes (|FC|≥ 1.3, FDR ≤ 1%) as a function of the simulated PMI revealed two significant clusters of 1427 of the genes (p < 0.001, r > 0.95, n = 7). AlegroMcode software predicted the presence of two kernels, one containing 474 genes expressed by glia represented in red and another of 317 genes predicted to be expressed by neurons. No differentiation between astroglia and microglia could be made in the glial gene cluster. Each node (circle) represents a gene and the links between nodes represents a significant correlation. The thickness and length of each link is proportional to the correlation value. (**B**) While neuronal gene expression decreased as function of time, glial gene expression increased. Stable genes are composed of 6754 genes that did not show any significant increase or decrease of expression during the simulated PMI. The majority (between 75 and 89%) of commonly used housekeeping genes are stable until 12 H then showed an increase of variability at the 24 H time point. Error bars on glial and neuronal cells correspond to the weighted standard error of the mean. The error bar for the stable and housekeeping genes corresponds to the standard deviation. Gene expression values are normalized to expression at time 0. (**C**) The 500 activity-dependent genes that were most downregulated in healthy post-mortem brain were used to separate the 7 time points of the simulated PMI by PCA. The first component projection of the PCA has a value of 55%, its projection on the x-axis show a separation of the time points in order of 0H, 2H, 1H, 8H, 4H, 12H and 24H. No other meaningful separation was found on other components. (**D**) The quality of the RNA, assessed by the commonly used RIN number and gel electrophoresis, showed no clear signs of degradation (Supplementary_Material_S1)**.**

One possible explanation for the selective loss of activity-dependent genes could relate to the stability of various cell populations during the simulated PMI. As a means to implicate specific cell populations that could be responsible for the reduction of genes during the simulated PMI we used a clustering algorithm as we have previously described^9^. We found that 1427 genes (71% known brain activity-dependent genes) could be clustered across the seven time points of the simulated PMI. For these clusters, we used AllegroMcode to identify two main clusters. One cluster of 317 rapidly declining genes was predicted to be neuronal and strongly overlapped with the activity-dependent genes. A second cluster of 474 genes was predicted to be glial, including astrocytes and microglia (Fig. 3A). Remarkably, as the neuronal cell cluster rapidly fell, there was a reciprocal and dramatic increase in the expression of the glial cell cluster (Fig. 3B). PCA on all 7 time points of the simulated PMI using the 500 most downregulated activity-dependent genes in postmortem samples revealed the same type of separation from the time point 0H to the latest time point 24H (Fig. 3C). Gene ontology analysis of the genes composing the two main clusters using the whole human genome as reference indicate mostly phagosome based pathways for glia cells while synapses appear to be the most significant pathway in the neuronal cell (Table 3).

**Table 3:** Gene ontology.

Glia	Neuron
Lysosome*** Phagosome*** Antigen processing and presentation*** MHC II*** & α/ß chain, N-terminal* Graft-versus-host disease** T-Cell activation*	Cell junction*** Synapse** Postsynaptic* Plasma membrane*

Using the Pathway tool DAVID V6.7 (Medium stringency, whole human genome as reference), the most represented pathways in the glia cells appear related to Phagocytose while the most represented pathways in Neuron cell are related to synapses.

***p < 1E−3. **p < 1E−2, *p < 5E−2.

Most studies assess RNA stability using a combination of RNA electrophoresis and housekeeping gene stability. Surprisingly, RNA stability at each time point showed no degradation except for a slight decrease of RNA quality at time point 1H and 4H using the commonly used RIN numbers and total RNA electrophoresis (Fig. 3D). The slight decrease of RNA integrity at time points 1H and 4H could explain the non-linearity of results observed at these two time points specially visible in the glia profile.

We further examined the expression of 64 reference genes (‘housekeeping' genes) commonly used in human genomic experiments for normalization across the simulated PMI (Supplemental_Table_S2.xlsx). From these 64 reference genes, only four were not stable, 48 (75%) were part of 6754 genes that were highly stable from 0 to 24 H, and 57 (89%) were stable until 12H. This corresponds to a ~ tenfold enrichment of housekeeping reference genes among the genes that were stable across the simulated PMI.

**Figure 4:**
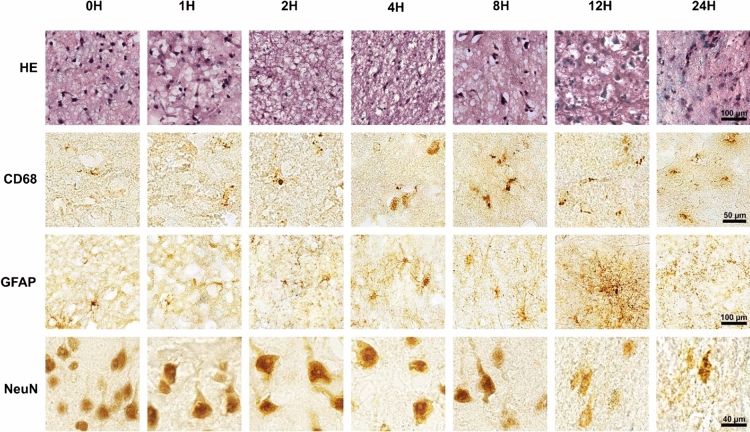
Loss of neuronal staining progressed while micro- and astro-glial processes greatly expanded during the simulated PMI. From 0 to 2H, glia in the gray matter were mainly non-reactive and neurons (NeuN) were degrading, starting at 4H and peaking at 12H the activation of microglia (CD68) and astrocytes (GFAP) was seen with overlapping cellular processes. Small GFAP positive nodules appeared on astrocytic processes at 8H and increased so that by 12H they became the most prominent form of GFAP staining. At 24H, tissues appeared physically degraded with astrocyte cell bodies no longer identifiable, neurons (NeuN) mostly degraded, and rounded microglia. (HE = hematoxylin & eosin staining).

In addition to performing RNAseq, at each time point a portion of each tissue sample was immediately placed in 4% paraformaldehyde for histological examination. We stained each block of tissue with H&E as well as specific markers for neurons (NeuN), microglia (CD68) and astrocytes (GFAP) and reviewed these with our neuropathologist (TVN) (Fig. 4). At 2 h, we observed a decrease of nuclear staining for NeuN and neuronal swelling. Between 4–8 h a majority of the neurons were swollen with a marked reduction in nuclear NeuN staining and by 12 h the neurons demonstrated loss of nuclear detail on H&E and NeuN staining was markedly reduced. At 24 h staining by NeuN showed that a majority of neurons were degraded. In contrast, between 2–4 h microglia became activated with increased process outgrowth peaking at 12 h. In parallel, astrocytes stained with GFAP remained small and non-reactive until 4 h with non-overlapping processes after which GFAP showed a highly heterogeneous staining pattern suggesting that some astrocytes were undergoing an outgrowth of processes that continued through 12 h. At 24 H, small GFAP-positive nodules measuring roughly 5 µm in diameter were seen and astrocyte cell bodies were no longer identifiable compatible with protein degradation observed in previous studies^22^.

## Discussion

Experiments on the human brain are critical for understanding and developing treatments for neurological and psychiatric disorders, especially because of the relatively poor translatability from animal models. Most studies on the human brain are performed after death with an average postmortem interval of more than 12 h^23^. Fresh human cortex, while relatively rare, can be obtained from human brain disorders requiring brain surgery, such as epilepsy and brain tumors. These fresh human brain tissues offer a unique opportunity to study transcription without worrying about RNA degradation in the postmortem interval. Here, we compared fresh versus postmortem measures of RNA transcription and found remarkable differences in transcriptional patterns between fresh and postmortem brain. There were striking and selective changes in the expression of brain activity-dependent transcripts out of proportion to other genes. This did not appear to be epilepsy disease-specific and was seen in both normal individuals and patients with Schizophrenia, Autism, Parkinson’s, and Huntington’s disorders. They were not age-specific since we have seen these genes present from age 1 to 56 years^9^. Activity-dependent neuronal genes are likely of critical importance in the pathophysiology of many of these brain disorders, thus their selective loss in the postmortem period could have a significant impact on the interpretation of genomic studies on postmortem human brain for many neuropsychiatric disorders.

Measurements of splicing complexity were also vastly reduced in postmortem brain tissues for some key genes notably the *RBFOX1* gene. The complete gene expression of freshly isolated human brain samples allows an unprecedented view of the genomic complexity of the human brain because of the preservation of so many different transcripts no longer present in postmortem tissues. Among brain genes evolutionary conserved, we found ultracomplex genes that showed thousands of different transcripts, widely shared through the evolutionary process, even with fruit flies. In the human brain, previous bioinformatic studies comparing alternatively spliced transcripts found that this startling complexity of alternative splicing^24^ is potentially linked to a highly conserved maintenance of neuronal function, critical for survival.

To have a more accurate understanding of the difference between fresh brain tissues and time-dependent postmortem tissues, we examined human brain gene expression and histology as a function of time after surgical removal to mimic the effect of the PMI. In human brain banks, while many strive for reducing the postmortem interval, tissues used for genomic analysis have had an average PMI of 18 ± 10 h^23^. It is well known that removal of brain tissues from their normal environment can lead to rapid death of neurons once blood circulation is no longer available to oxygenate the tissue^25^. Thus it is not surprising that many of the changes we have seen in our ‘simulated death' experiment can be seen after hypoxic brain injury with reduced neuronal gene expression and increased astrocytic gene expression^26^.

During the PMI, the time to get the body down to 4 °C is an important measure for RNA stability and a major factor in the degradation of the proteome^27^. To date, no accurate model of postmortem human brain temperature has been validated, but some have assumed a reduction in 1 °C per hour for the first 12 h immediately after death followed by 0.5 °C per hour thereafter^28^. It can take up to 30 h for the human brain to cool even when the body is refrigerated^29^. Therefore, while we studied the effect of simulated PMI at room temperature, in most cases following death the brain will be at a much higher temperature for a longer period than we used in our study here and therefore would likely have more rapid changes. While some studies have suggested that the RNA transcriptome is stable up to 30 h after death^23,30^, dynamic changes in RNA levels for specific cell types described here as a result of the PMI have been reported by others showing RNA degradation^31^, chromatin modification^6^, activation of gene expression^32–34^, and protein degradation^22^. While we did not directly look at protein expression, the predicted cell differences implicating specific cell populations would likely create corresponding proteomic changes in glia and neuronal cell populations. Here we used histological measures to confirm that selective changes in RNA expression come from glial and neuronal cell population changes. Previous studies have demonstrated neurodegeneration starting at a PMI of 4.5 h and have shown decreased NeuN on Western blots with relative preservation of housekeeping genes^35^. In rodents, a PMI of 6 h was associated with significant changes in gene expression that could strongly influence the interpretation of studies^33,36,37^. In addition, pig brain neurons, that have a striking similarity with human neurons^38^, show atrophy and disintegration somewhere between 1 and 10 h after death^39^.

We used transcriptional clustering to predict and histology to confirm cell-specific changes as a function of time of the simulated PMI. There was a remarkable increase in glial specific genes and glial processes that paralleled reductions in neuronal genes and neuronal cell integrity. The rapid loss of activity-dependent genes is likely related to their known biological functions. Many genes involved in brain activity, particularly pathways of learning and memory, are short-lived and purposefully transient in their expression as a part of their important functions in differentiation acute versus long-term brain changes needed for memory consolidation^40^. Similarly, the pronounced, time-dependent activation of microglia and astrocytes is likely due to their normal roles in brain homeostasis and reactions to low oxygen levels as seen after stroke or hypoxic brain injury.

Other studies have also shown that the RIN (RNA Integrity Number) and gel electrophoresis, commonly used measures of RNA stability based on the integrity of rRNA, can be relatively stable for up to 24H indicating that random RNA degradation by RNases occurs at a rate too low to have any significant effect during commonly used PMI^30,41^. Similarly, our RIN values did not change over 24H indicating that the RNAs with decreased or increased expression were due to ongoing biological processes^42^. Using RNA stability by electrophoresis is thus not a reliable measure of tissue stability. A more targeted measure of transcriptional stability could be made that focuses on cell-type specific changes of genes with relatively short half-lives in the PMI.

Similar to the RIN, many genes were surprisingly stable in our analyses. Some of the most stable of these are ‘housekeeping’ genes that have been historically used as references for RNA stability and normalization of RNA expression levels from a large number of different cell types and conditions^43^. In fact, the total amount of RNA per mg of tissue was not reduced in our studies. This may in part be due to our observation that the reduction of neuronal genes was mostly compensated by a parallel increase of glia gene expression. Therefore, the use of housekeeping genes or the total amount of RNA used as references for normalization of gene in tissues with a simulated PMI greater than 4 h will not be able to detect the bias favoring glia gene expression over neuronal genes and will produce significant bias in genomic quantitative studies when comparing brain tissues from patients with different PMIs. Along these same lines, any postmortem studies focused on neuronal or glial mechanisms, especially in brain disorders where cognition and behavior are important, will be significantly impacted by the PMI. False positive and negative results related to immune gene expression will similarly be present in postmortem studies due to postmortem activation of microglia and astrocytes when the PMI is 4 h or more.

In summary, our findings raise awareness of the exceptional value of fresh human brain tissues for genomic studies as well as a need to apply additional scrutiny to interpret results from human postmortem brain studies. Genome-wide measures of RNA stability as a function of the PMI from studies such as the one here could in fact be used to determine and perhaps correct for the stability of individual genes and exons as a function of time and temperature during the PMI^27^. This correction would need to be combined with knowledge about the cause and timing of death, since ongoing changes such as hypoxia prior to death could also play a significant role in the specific RNAs^28,35^.

## Methods

### Brain activity-dependent genes derived from human epilepsy patient surgical resections

We define ‘brain activity-dependent' genes as a list of genes related to high versus low epileptic brain activity measured by intracranial recordings. The brain samples used to build this list were taken from epileptic patients who have undergone surgery for their epilepsy. In this clinical operation, the patients have implanted electrodes that measure the degree of epileptic and activities at each location^9^. A key advantage of our study design was that highly active brain regions are compared first to less active, internally controlled, samples from the same patient and then common changes are identified across many patients. This focuses on transcriptional effects of brain activity genes that were globally in common across all patients. This study design excludes differences in sex, age, medications, genetic background, and tissue processing. We performed this research on cortical tissues not needed for clinical care or diagnosis that were removed as part of epilepsy surgery for 20 patients. Informed consent was obtained from each patient (or legal guardian/parent for subjects under 18) at Wayne State University (Detroit. MI, USA) and the University of Illinois (Chicago, IL) with IRB approvals at both institutions. All methods were performed in accordance with the relevant guidelines and regulations. Resected tissues were immediately placed on ice followed by freezing within 60 min at − 80 °C to protect RNA from degradation or *de-novo* transcription. For these studies, differential gene expression differences between more and less active brain regions were determined both using RNAseq and microarrays^7^. We cross-referenced the microarrays genes with the genes present in the RNAseq study and used a total of 15,655 genes that were both present in microarrays and in RNAseq experiments.

### RNAseq and PCA analysis

RNAseq was performed on two samples, one with low activity and one with high activity, from two epileptic patients (EP158 and EP168). Total RNA was prepared using TRIzol (Invitrogen) followed by DNase treatment, purified on an RNeasy column (Qiagen) as previously described^11^ and quantified both by nanodrop and Quibit. RNA was fragmented (Ambion Fragmentation reagents AM8740) and the quality was assessed using a Bioanalyser (Agilent). Poly A + paired-end stranded sequencing libraries were made using the Illumina TruSeq stranded sample preparation kit (Catalog No.15031048) as previously described^11^. Libraries were sequenced on the HiSeq platform using paired-end 100 bp chemistry. Sequences are available from the Short Read Archive (http://www.ncbi.nlm.nih.gov/sra).

RNAseq results obtained from our fresh samples were compared to RNAseq results of cortical brain regions from postmortem samples from 4 control patients without neurological disorders^10^, and from individuals with neuropsychiatric disorders: Schizophrenia^20^, Parkinson’s disease^19^, Huntington’s disease^10^ and autism disorder^18^ (Table 1). Differential expression was assessed using the software Qualimap 2 (V2.2^44^) using the default parameters and visualized with the software IGV (V2.3.68^45^). All the samples were part of the same pipeline of analysis, FPKM (Fragment per kb per Million reads) were quantile normalized between samples and had similar quality-check metrics.

The significance used for differential expression was |Fold Change|≥ 1.5 with a FDR ≤ 12%. Hypergeometric analysis was performed with the statistical software ‘R'. We separated the samples by PCA using the expression of 500 brain activity-dependent genes that showed the greatest decrease in postmortem samples. The barycenter of the clouds and the 80% confidence ellipses were computed using "FactoMineR"^46^.

### Fresh brain sample collection and RNA extraction

A freshly isolated region of human neocortex was obtained from a temporal lobe epilepsy adult, female patient and the histology of the tissue was reported to be non-pathological by a neuropathologist (TVN). At time zero, a portion of the neocortical tissue was frozen on dry ice (0H). The remaining tissue was placed at 24 °C and sealed in plastic wrap. At each designated time point (1, 2, 4, 8, 12 and 24 h), an additional sample was removed from the tissue block and cut in half. We did 7 dissections from the same tissue block, one half was fixed in 4% paraformaldehyde for 28 h for histological analysis and the other half was frozen on dry ice and stored at − 80 °C prior to RNA extraction. For RNA preparation, gray matter containing approximately equal amounts of each layer of the 6-layered cortex was used. Total RNA from each time point was isolated using Qiagen RNAeasy columns (mini kit, RNeasy lipid kit, Qiagen) following the manufacturer’s protocol.

### Statistical analysis

The reads were trimmed using ‘Trim Galore!' (Babraham Institute, Cambridge, UK) with default parameters resulting in a total of around 20 million reads for each sample. STAR (V2.7.0)^47^ was used for the alignment using GenCode V30 as reference resulting in an average of 90% of unique mappings. Gene counts were extracted using ‘SeqMonk' (Babraham Institute, Cambridge, UK). We used a statistical clustering algorithm to predict cell-type specific clusters as we have described^9^. In brief, this algorithm cross-references groups of brain activity-dependent genes that follow the same expression profile and clusters them using Pearson correlation metrics. These clusters are visualized using Cytoscape and identified using AlegroMcode (AllegroViva Corporation, 2011), cell assignation has been done using Tissues 2.0^48^ and corresponds to cells that are most likely to express them. Enrichments and depletions were computed with ‘R' by hypergeometric analysis^49^. A list of 65 human reference genes were taken from seven publications^15,16,50–54^.

### Immunohistochemistry

Immunohistochemistry and tissue staining (Hematoxylin & Eosin) were performed on fixed tissues at each time point that RNAseq data was obtained (0 to 24H). We used the following antibodies: Astrocytes GFAP (clone 6F2, Dako), Microglia CD68 (clone KP1, Dako) and Neurons with NeuN (cat #MAB377, Millipore). Signals were amplified using the ‘ABC kit' (Vectastain, Vector Labs) following the manufacturer’s protocol followed by developing with ‘SigmaFast DAB' (Millipore).

## Supplementary Information

Supplementary Information 1.

Supplementary Information 2.

Supplementary Information 3.
