# Supplementary figures and images for: Selective time-dependent changes in activity and cell-specific gene expression in human postmortem brain
Source: Sci Rep. 2021 Mar 23;11:6078. doi: 10.1038/s41598-021-85801-6 (PMC7988150; doi:10.1038/s41598-021-85801-6)

## Slide 1
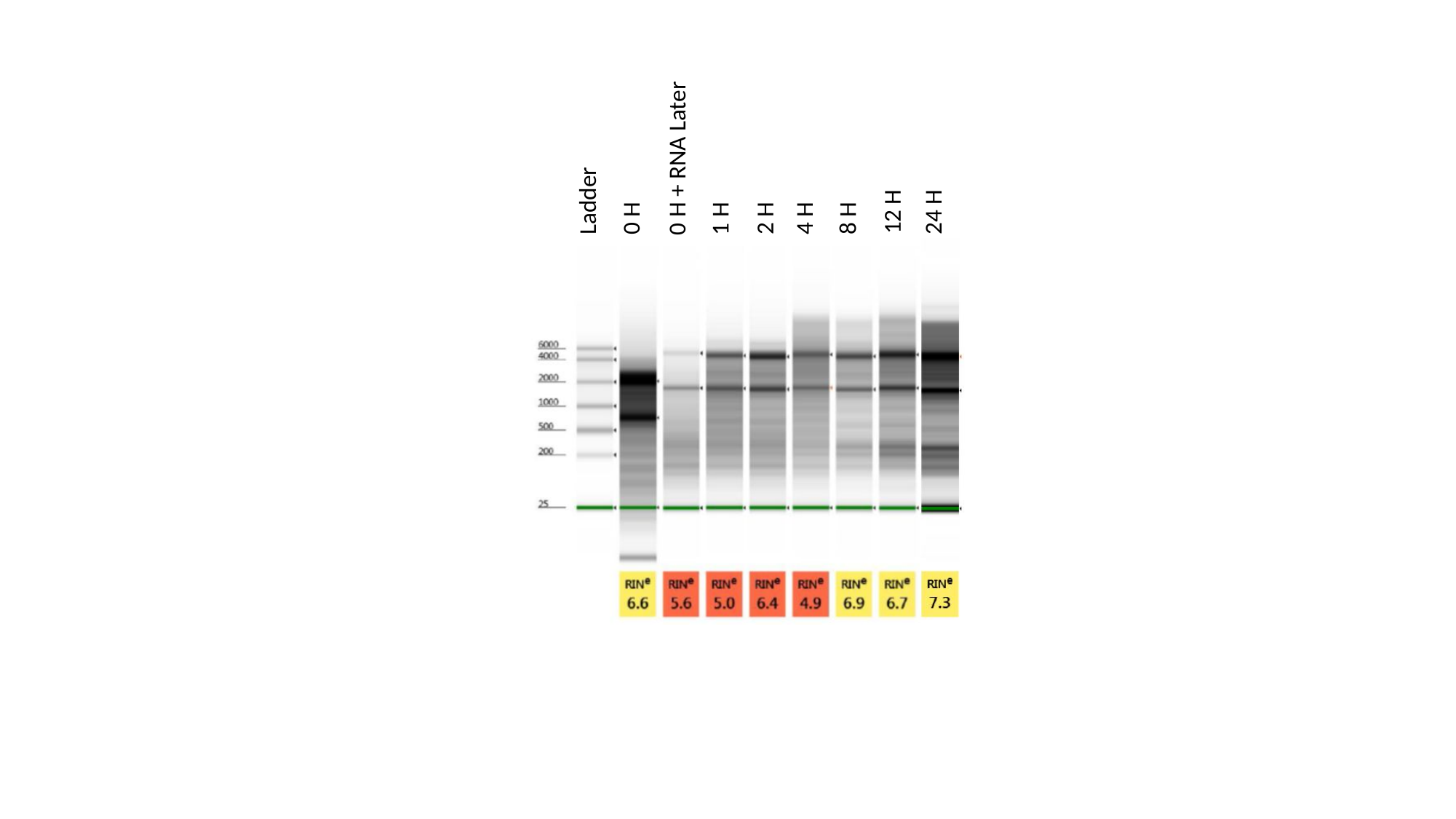

0 H + RNA Later
Ladder
12 H
24 H
4 H
8 H
2 H
0 H
1 H

## Slide 2
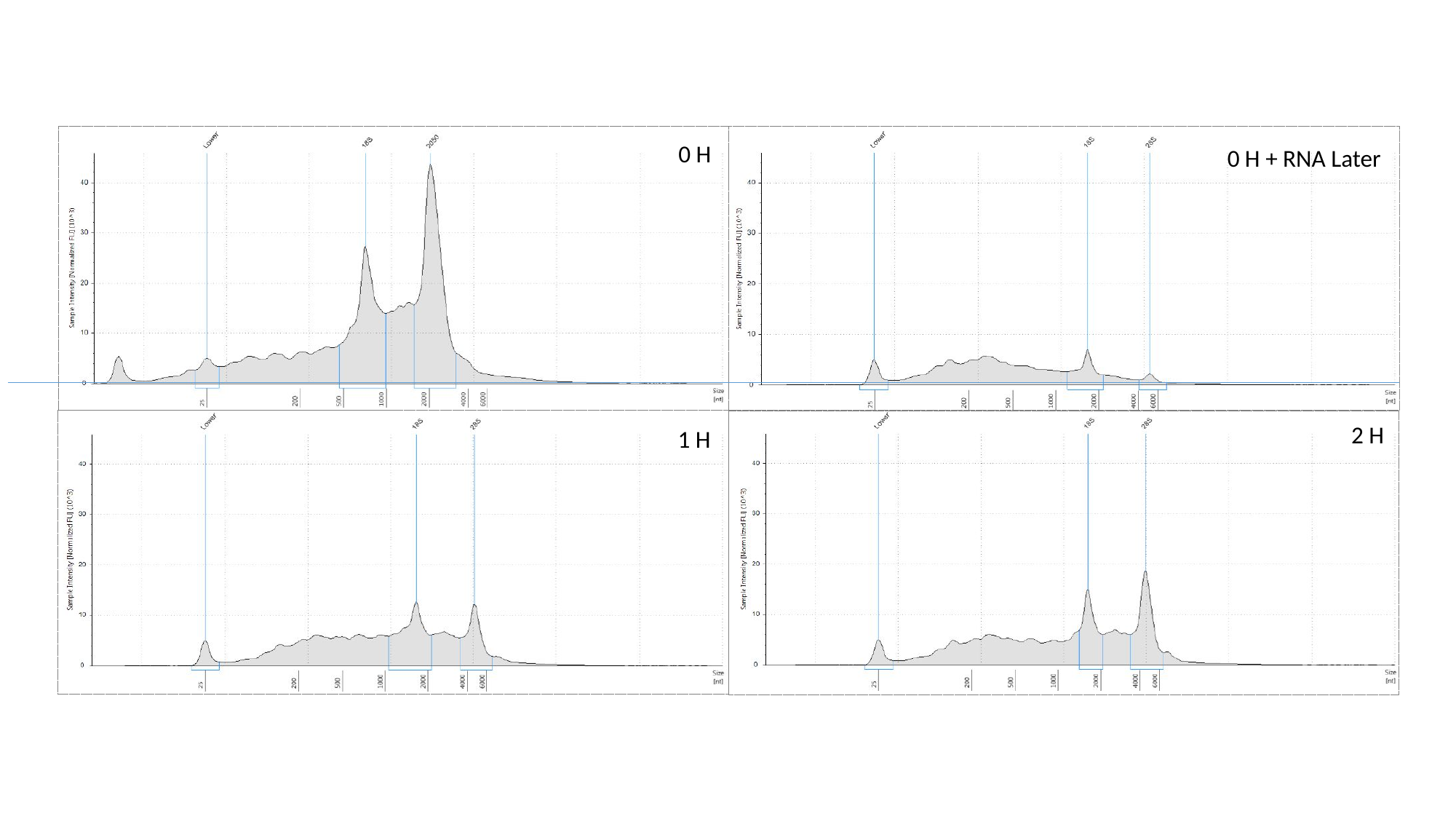

0 H
0 H + RNA Later
2 H
1 H

## Slide 3
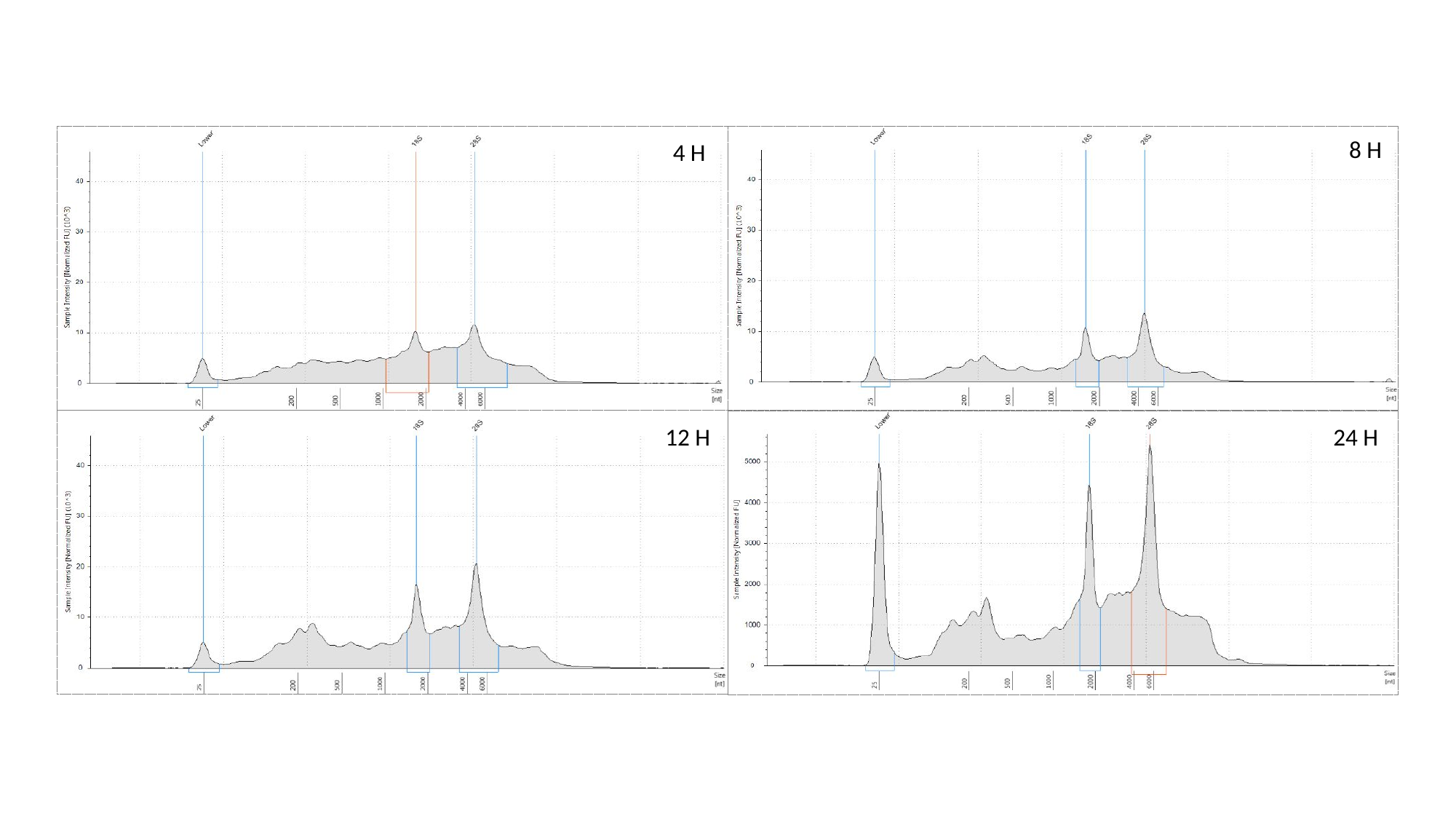

8 H
4 H
12 H
24 H

Supplement: Supplementary file 3 — Supplementary Information 3. [file 41598_2021_85801_MOESM3_ESM.pptx]
